# Globe Rupture – A Case Report and Review of Emergency Department Diagnosis and Management

**DOI:** 10.7759/cureus.30007

**Published:** 2022-10-06

**Authors:** Stephen Guilherme, Lisa O Iyeke, Tiana L Vazquez, Ravneet S Rai, Mark Richman

**Affiliations:** 1 Emergency Department, Northwell Long Island Jewish Medical Center, New York, USA; 2 Ophthalmology, Northwell Long Island Jewish Medical Center, New York, USA

**Keywords:** blunt globe injury, globe rupture diagnosis, ocular trauma, surgical enucleation, open globe rupture

## Abstract

Globe ruptures, while uncommon, must be promptly recognized to optimize the possibility of preserving vision. Differentiating open globe injuries from corneal abrasions, hyphema, and other ocular injuries is critical. When a globe rupture is identified, prompt ophthalmology consultation, placement of a rigid eye shield, administration of prophylactic systemic antibiotics (and antifungals with an appropriate history), and a tetanus vaccine update (if needed) are key to a successful outcome, so long as the visual prognosis is not dismal. We describe a case of ocular trauma presenting as a blood blister adherent to the cornea, which was initially assessed (by the triage nurse) to be a blood clot but later identified as a globe rupture. We include a discussion of globe rupture recognition and its management by the emergency department.

## Introduction

Ocular trauma is a leading cause of monocular blindness in the United States [1], with globe rupture or open globe injury being one of the greatest risk factors for trauma-related blindness [2]. "Globe rupture" is the common term used to describe all types of open globe injuries (e.g., globe laceration or perforation). Open globe injuries are full-thickness wounds of the cornea or sclera that are often caused by laceration or blunt force trauma to the eye [3]. Globe ruptures occur at a rate of three per 100,000 people annually (9,000 cases per year in the U.S.) [4]. Common causes of blunt globe injuries are rocks, fists, baseballs, lumber, and fishing weights; penetrating injuries are often caused by paintballs and BB (ball-bearing or bullet ball) guns [5]. Falls are a major contributor to the occurrence of globe ruptures in the elderly [6]. The Ocular Trauma Score (OTS) provides a system that can be used to predict the final visual outcome of an injured eye and has 95% sensitivity, predicting the return of light perception or better, and a 100% prediction of no light perception [7].

## Case presentation

An 85-year-old woman with a past medical history of osteoarthritis, hypertension, type 2 diabetes, hyperparathyroidism (status post parathyroidectomy), and presbyopia presented with a red globule protruding from the front of her right eye after a fall from her couch. The patient was wearing corrective lenses at the time of the fall, and the corrective lenses were not damaged. The patient was unsure of what struck their globe during the fall. The patient reported clear fluid exiting the eye, followed by swelling and bleeding. The patient had a complete loss of vision and expressed feelings of pressure around the injured eye.

**Figure 1:**
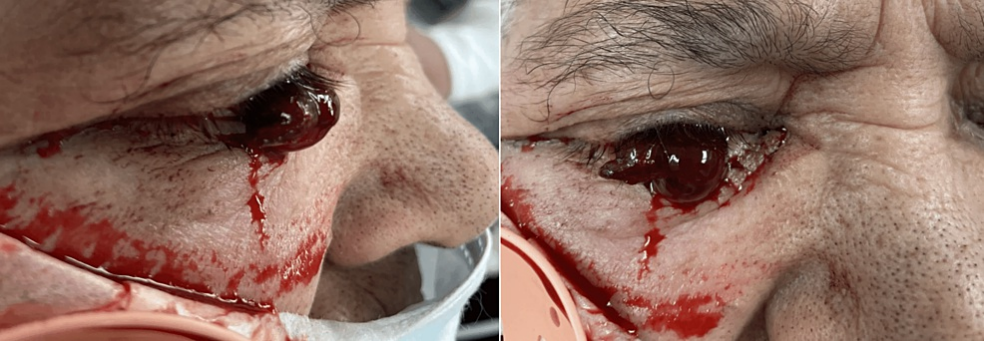
Lateral and anteroposterior views of the patient's eye upon presentation.

The emergency medicine physician was called by the triage nurse to determine whether the nurse should wipe the "clot" away from the front of the patient’s eye. The physician noted the following physical examination findings: the right eye had no light perception, a 3+ injection, and a large corneal laceration with uveal prolapse (Figure 1).

**Figure 2:**
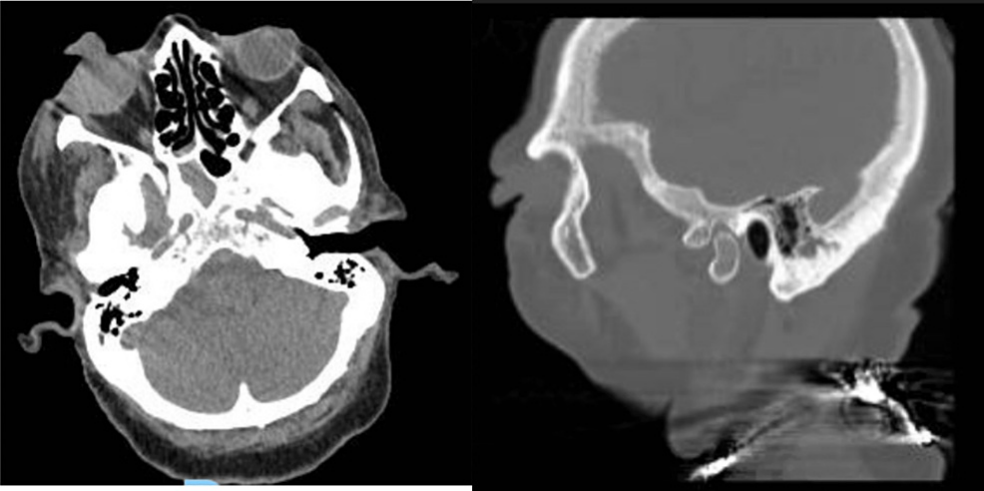
CT of the brain (transverse section) demonstrates prolapsed uveal contents of the right eye (left). CT maxillofacial (sagittal section) demonstrates prolapsed uveal contents of the right eye (right).

Based on the history and exam, there was a clinical concern for a diagnosis of globe rupture. An eye shield was placed, and vancomycin, ceftazidime, and tetanus vaccine were administered. Computed tomography (CT) of the brain showed a small soft tissue nodule along the anterior aspect of the globe, soft tissue swelling on the right side of the face, no significant retrobulbar hemorrhage, and no fractures, while CT of the orbits did not reveal an intraocular foreign body or retrobulbar hematoma (Figure 2).

Ophthalmology was consulted, and the patient’s vision was deemed unsalvageable. The patient subsequently underwent enucleation of the affected eye.

## Discussion

A globe rupture is defined by the Birmingham Eye Trauma Terminology (BETT) as a full-thickness injury of the eyewall by a blunt object and requires emergent diagnosis and treatment. Physical examination findings such as a teardrop pupil or flattened globe should increase suspicion of a globe rupture and prompt immediate ophthalmology consultation. An eye shield should be used to protect the affected eye(s), and a tetanus shot should be given if the patient has not had one in the last five years. Antibiotics with methicillin-resistant Staphylococcus aureus (MRSA) and Pseudomonas coverage should be administered (e.g., vancomycin and ceftazidime; in the event of allergies, clindamycin and gentamicin are alternative agents). In cases that are high risk for endophthalmitis, such as gross contamination (such as from soil) or an effusion around a foreign body, intravitreal antibiotics (e.g., vancomycin and ceftazidime) can be used [8]. Permanent blindness, chronic pain, endophthalmitis, retained foreign bodies, secondary cataract, belted corneal degeneration, iris atrophy, and/or an irregular pupil can all result from a globe rupture. If the visual prognosis is poor, enucleation may be needed to prevent sympathetic ophthalmia (a bilateral, granulomatous, autoimmune inflammatory response uveitis that may cause blindness in the opposite eye) [9].

The differential diagnosis of globe rupture includes corneal abrasion, corneal ulceration, subconjunctival hemorrhage, hyphema, orbital blowout fracture, traumatic iritis, and retrobulbar hematoma. In subtle cases, the most difficult and important task is to distinguish between corneal abrasion and globe penetration. The physical examination finding of a flattened globe or a positive Seidel sign (aqueous humor leakage from the anterior chamber after applying fluorescein staining to the lower conjunctival sac) indicates globe rupture and negates corneal abrasion. Less obvious cases of rupture might only manifest with a teardrop-shaped pupil and/or positive Seidel sign, and a slit lamp exam can be useful in detecting subtle full-thickness corneal laceration and penetration.

If globe rupture is suspected, no pressure should be placed on the eye to reduce the risk of further extravasation of intraocular contents. Therefore, ocular pressure should not be obtained. In all cases of suspected globe rupture, a CT of the orbits (with thin (1 mm) cuts in the axial, coronal, and sagittal planes) should be performed. No ocular ultrasound should be performed to avoid an increase in ocular pressure. No MRI should be performed if there is suspicion of an intraocular foreign body or if metal may have caused the globe injury.

Globe penetration or rupture not requiring enucleation should be repaired surgically. Primary enucleation should be avoided until the severity of ocular trauma is established. Patients with no light perception (NLP) prior to surgery have a 15-20% chance of improving to light perception (LP) after surgery [5]. Therefore, enucleation should be avoided shortly after presentation unless the visual prognosis is dismal. Surgical repair or enucleation should occur as early as possible, within two weeks of injury, to decrease the risk of sympathetic ophthalmia [7]. If repair is performed and analgesia or anesthesia is necessary, ketamine and succinylcholine should not be used, as these may increase intraocular pressure.

## Conclusions

Patients suspected of globe rupture must be urgently examined and investigated via ophthalmological consultation, careful anterior examination, and a CT scan to confirm the diagnosis. Once confirmed, a shield cover should be placed, ophthalmology should be consulted, and antibiotics and a tetanus vaccine should be administered, if necessary. Ocular ultrasound and MRI should be avoided. As open globe injuries may occur in the context of other trauma, if sedation or analgesia are required, ketamine and succinylcholine should be avoided.
